# Does Combination Therapy With SGLT2 Inhibitors and Renin–Angiotensin System Blockers Lead to Greater Reduction in Cardiorenal Events Among Patients With Type 2 Diabetes?

**DOI:** 10.3389/fcvm.2021.679124

**Published:** 2021-05-05

**Authors:** Li-Min Zhao, Miao Zhang, Ze-Lin Zhan, Mei Qiu

**Affiliations:** ^1^Department of Endocrinology, Shenzhen Longhua District Central Hospital, Shenzhen, China; ^2^Department of Nephrology, Shenzhen Hospital of Beijing University of Chinese Medicine, Shenzhen, China; ^3^Class 3, Clinical Medicine, Grade 2019, The Second Clinical Medical College, Southern Medical University, Guangzhou, China; ^4^Department of General Medicine, Shenzhen Longhua District Central Hospital, Shenzhen, China

**Keywords:** SGLT2 inhibitors, RAS blockers, type 2 diabetes mellitus, cardiorenal events, Heart failure

## Introduction

We have read with great interest a meta-analysis (1) entitled “Efficacy and safety of Combination Therapy with Sodium-glucose Transporter 2 Inhibitors and Renin-Angiotensin System Blockers in Patients with Type 2 Diabetes: A Systematic Review and Meta-Analysis” conducted by Tian et al. In that meta-analysis (1), Tian and colleagues identified that, compared with the monotherapy of angiotensin-converting enzyme inhibitors (ACEIs) or angiotensin receptor blockers (ARBs), the combination therapy with sodium-glucose cotransporter 2 (SGLT2) inhibitors and ACEI/ARB led to greater improvement in some of the intermediate indicators such as blood pressure, blood glucose, and estimated glomerular filtration rate (eGFR) among patients with type 2 diabetes mellitus (T2DM). However, the authors failed to assess any of the final outcomes such as cardiovascular events and renal events. Because whether the combination therapy above has superiority over monotherapy in lowering cardiorenal events means a lot to the selection between them, we carried out this meta-analysis based on the cardiovascular or renal outcome trials (CVOTs) assessing gliflozins in T2DM patients.

## Methods and Findings

Three cardiorenal endpoints of interest were major adverse cardiovascular events (MACEs), cardiovascular death or hospitalization for heart failure (CV death or HHF), and composite kidney outcome (CKO). MACE was a composite of cardiovascular death, non-fatal stroke, or non-fatal myocardial infarction. CKO was a composite of sustained 40% reduction in eGFR or doubling of serum creatinine, end-stage kidney disease or initiation of renal-replacement therapy, or renal death. By searching PubMed, Embase, and Cochrane Central Register of Controlled Trials (CENTRAL) and then performing study selection, we finally included seven CVOTs (2–8) of gliflozins enrolling T2DM patients. All the patients enrolled in the CREDENCE trial (2) received a stable dose of an ACEI or ARB, and therefore, the data of all the patients in that trial (2) were included in our analysis. The four trials of VERTIS CV (3), CANVAS Program (4), DECLARE-TIMI58 (5), and EMPA-REG OUTCOME (6) provided the data of the subgroup of patients receiving an ACEI or ARB, and therefore, those subgroup data were included in our analysis. Since most of the participants in the SOLOIST-WHF (7) and SCORED (8) trials received an ACEI or ARB, the outcome data based on the whole participants in the two trials (7, 8) were incorporated in our analysis. We conducted both random-effects and fixed-effects meta-analysis with the inverse variance method, using the data of hazard ratios (HRs) and 95% confidence intervals (CIs) extracted from included articles. If *I*^2^ > 50% (meaning substantial heterogeneity), we would use the results of random-effects meta-analysis. Otherwise, we would use the results of fixed-effects meta-analysis. A sensitivity analysis excluding the SOLOIST-WHF (7) and SCORED (8) trials was done. Publication bias detection was done by funnel plots and Egger test. All the data analyses were implemented using the Stata/MP 16.0.

**Figure 1:**
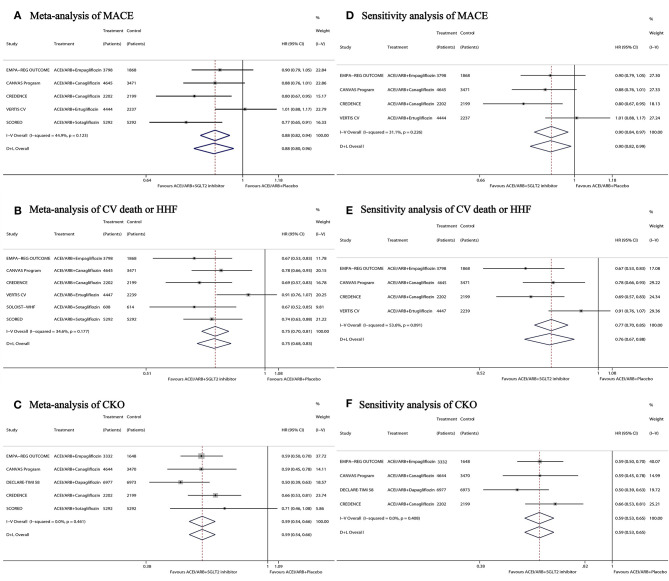
Meta-analysis **(A**–**C)** and sensitivity analysis **(D**–**F)** of ACEI/ARB + SGLT2 inhibitor vs. ACEI/ARB + placebo on MACE, CV death or HHF, and CKO. ACEI, angiotensin-converting enzyme inhibitors; ARB, angiotensin receptor blockers; SGLT2, sodium-glucose transporter 2; MACE, major adverse cardiovascular events; CV death or HHF, cardiovascular death or hospitalization for heart failure; CKO, composite kidney outcome; HR, hazard ratio; CI, confidence interval.

Compared with ACEI/ARB + placebo, ACEI/ARB + SGLT2 inhibitor led to a greater reduction in the risk of MACE (HR 0.88, 95% CI 0.82–0.94; *I*^2^ = 44.9%; Figure 1A), CV death or HHF (HR 0.75, 95% CI 0.70–0.81; *I*^2^ = 34.6%; Figure 1B), and CKO (HR 0.59, 95% CI 0.54–0.66; *I*^2^ = 0%; Figure 1C). The results of sensitivity analysis showed a similar reduction with ACEI/ARB + SGLT2 inhibitor in the risk of MACE (HR 0.90, 95% CI 0.84–0.97; *I*^2^ = 31.1%; Figure 1D), CV death or HHF (HR 0.76, 95% CI 0.67–0.88; *I*^2^ = 53.6%; Figure 1E), and CKO (HR 0.59, 95% CI 0.530.65; *I*^2^ = 0%; Figure 1F) vs. ACEI/ARB + placebo. Funnel plots and the Egger test results (Supplementary Figure 1) did not suggest any publication bias in terms of any of the three outcomes.

## Discussion

This meta-analysis is the first one that has demonstrated the greater efficacy with the combination therapy of ACEI/ARB and SGLT2 inhibitor in lowering MACE, CV death or HHF, and CKO compared to the ACEI/ARB monotherapy among T2DM patients. The relative superiority of the combination therapy over the monotherapy on the three final outcomes is supported by that on those intermediate indicators such as blood pressure and eGFR, as revealed in Tian et al.'s meta-analysis (1).

As we all know, ACEI/ARB is able to improve cardiovascular and renal prognosis. Thus, this drug class is widely licensed for the treatment of chronic heart failure (HF), primary hypertension, T2DM with chronic kidney disease (CKD), etc., as long as there are no relevant contraindications. Meanwhile, increasing meta-analyses (9–12) assessing the cardiorenal efficacy of gliflozins in T2DM patients have confirmed the evident efficacy with gliflozins vs. placebo in lowering death and cardiorenal events. Thus, there is an urgent need to know whether the combination therapy of ACEI/ARB and gliflozins can produce greater reduction in the incidence of cardiorenal events than the monotherapy of ACEI/ARB or gliflozins can. Our meta-analysis revealed that using ACEI/ARB and gliflozins together was more effective than using ACEI/ARB alone in lowering arteriosclerotic cardiovascular (ASCV), HF, and renal failure events. This finding will guide the combination use of the two drug classes in T2DM patients at high risk of developing cardiorenal events, such as T2DM patients with ASCV disease, CKD, or HF.

This study has three main weaknesses. First, this is a study-level meta-analysis. Thus, we failed to consider the impact of baseline use of other cardiovascular or antihyperglycemic drugs on the efficacy of combination therapy assessed in the study. Future studies exploring the combination therapy of two or more relevant drugs are needed. Second, we failed to assess whether using ACEI/ARB and gliflozins together is more effective than using gliflozins alone. This issue requires further investigation. Third, this study reveals the superiority of ACEI/ARB + SGLT2 inhibitor over ACEI/ARB + placebo in reducing cardiovascular and renal events among patients previously receiving ACEI/ARB. However, whether the additive positive effect of ACEI/ARB and SGLT2 inhibitor exists in patients previously receiving neither ACEI/ARB nor SGLT2 inhibitor needs to be validated in future studies.

In conclusion, the combination therapy of gliflozins and ACEI/ARB is more effective than the ACEI/ARB monotherapy in preventing ASCV, HF, and renal failure events among patients with T2DM, which suggests that this kind of combination therapy should be recommended in T2DM patients, especially those with HF or CKD, for better prevention of cardiorenal events.

## Author Contributions

MQ: design. L-MZ, MZ, and Z-LZ: conduct/data collection. MZ and Z-LZ: analysis. L-MZ and MQ: writing manuscript. All authors contributed to the article and approved the submitted version.

## Conflict of Interest

The authors declare that the research was conducted in the absence of any commercial or financial relationships that could be construed as a potential conflict of interest.

## Supplementary Material

Supplementary Figure 1 Publication bias detection for MACE **(A)**, CV death or HHF **(B)**, and CKO **(C)**.
