# Supplementary figures and images for: Does Combination Therapy With SGLT2 Inhibitors and Renin–Angiotensin System Blockers Lead to Greater Reduction in Cardiorenal Events Among Patients With Type 2 Diabetes?
Source: Front Cardiovasc Med. 2021 May 5;8:679124. doi: 10.3389/fcvm.2021.679124 (PMC8131654; doi:10.3389/fcvm.2021.679124)

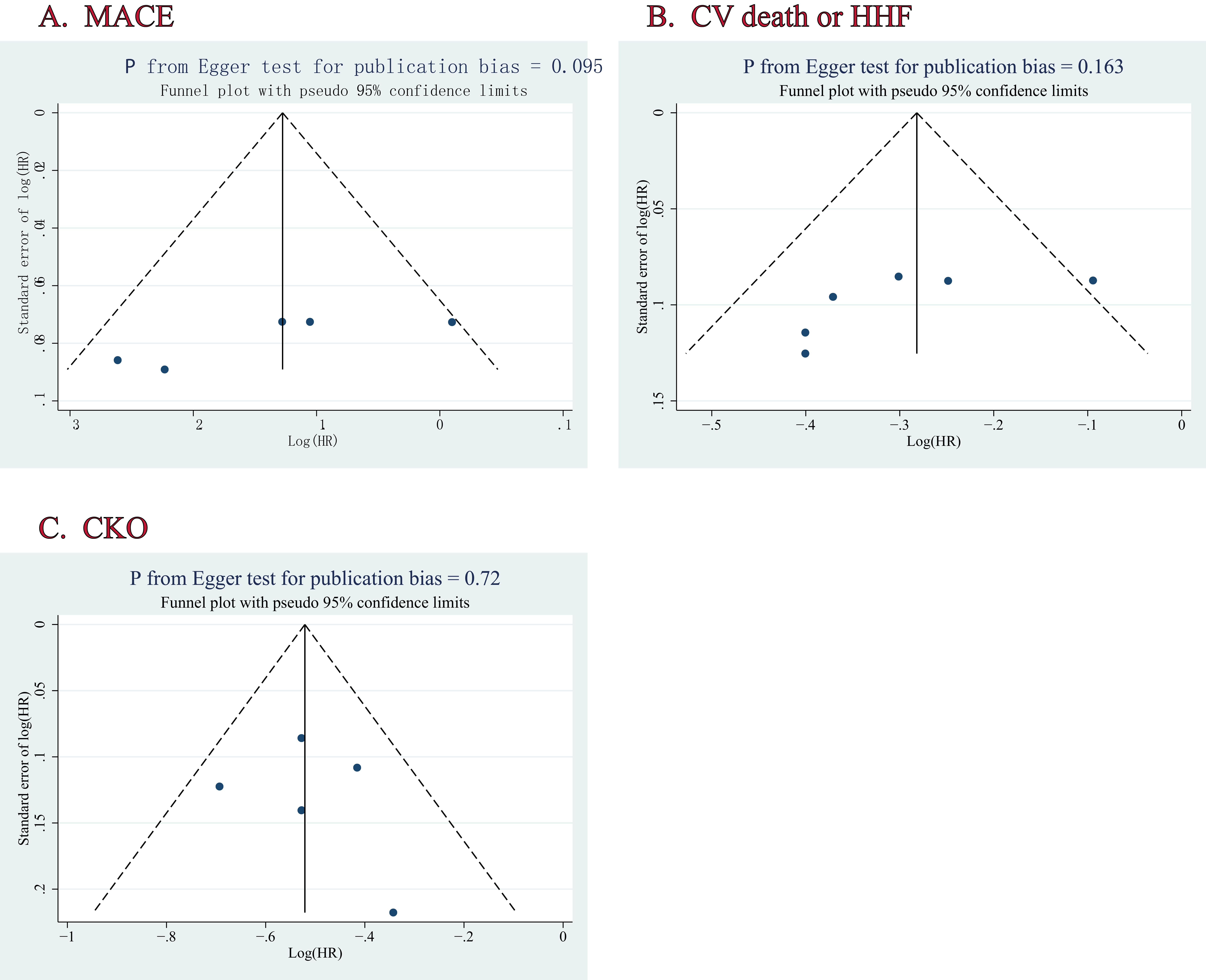

Supplement: Supplementary Figure 1 — Publication bias detection for MACE (A), CV death or HHF (B), and CKO (C). [file Image_1.JPEG]
